# Structural permeability of complex networks to control signals

**DOI:** 10.1038/ncomms9349

**Published:** 2015-09-22

**Authors:** Francesco Lo Iudice, Franco Garofalo, Francesco Sorrentino

**Affiliations:** 1 Department of Electrical Engineering and Information Technology, University of Naples Federico II, 80125 Naples, Italy; 2 Department of Mechanical Engineering, University of New Mexico, Albuqeurque, New Mexico 87131, USA

## Abstract

Many biological, social and technological systems can be described as complex networks. The goal of affecting their behaviour has motivated recent work focusing on the relationship between the network structure and its propensity to be controlled. While this work has provided insight into several relevant problems, a comprehensive approach to address partial and complete controllability of networks is still lacking. Here, we bridge this gap by developing a framework to maximize the diffusion of the control signals through a network, while taking into account physical and economic constraints that inevitably arise in applications. This approach allows us to introduce the network permeability, a unified metric of the propensity of a network to be controllable. The analysis of the permeability of several synthetic and real networks enables us to extract some structural features that deepen our quantitative understanding of the ease with which specific controllability requirements can be met.

## Abstract (web summary)

Understanding how to control complex networks can be useful to steer interconnected systems towards a desired state. Here, the authors introduce the concept of network permeability, a unified metric of the propensity of a network to be controllable taking into account physical and economic constrains.

## Graphical abstract

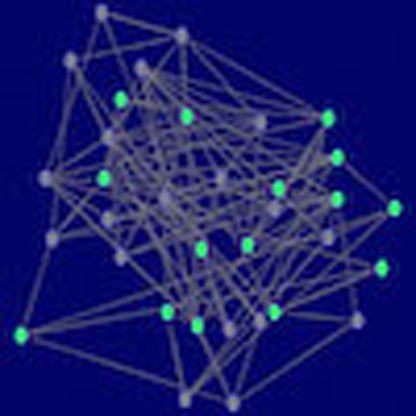

Complex networks have attracted considerable attention from the scientific community^1^,^2^,^3^,^4^,^5^,^6^,^7^ owing to their ubiquity in nature and in artificial settings. Whether these networks are natural or artificial, the question arises how mankind can control their behaviour. For instance, in pinning control^8^,^9^,^10^,^11^,^12^,^13^,^14^,^15^,^16^,^17^, a complex dynamical network can be controlled by injecting control signals in a limited number of nodes (the driver nodes), provided that these are chosen properly. As complete controllability is a required condition for pinning control, refs 8, 9, 10, 11, 12, 13, 14, 15, 16, 17 have dealt with and addressed complete controllability problems in the context of complex networks.

The problem of selecting the driver nodes so to ensure complete controllability of complex networks has been approached by following the geometrical mapping of Kalman's theory^18^ proposed by Lin^19^. Recent work has focused on analysing the conditions for complete controllability^20^,^21^,^22^,^23^,^24^,^25^. However, in applications, achieving complete controllability is often a chimera as both economical and physical constraints typically affect the selection of the driver nodes. For instance, previous work^20^,^26^,^27^ has pointed out that for gene regulatory networks, a considerable amount of driver nodes are needed to achieve complete controllability, which can turn out unfeasible. In applications, the problem often arises of finding the set of driver nodes ensuring controllability of another set of nodes. This is the case, for instance, when attempting to design curative interventions for cancer, as one is typically interested in acting only on cells lying in carcinogenic and pre-carcinogenic state^28^,^29^. Moreover, it is often the case that the selection of the driver nodes is restricted to a well-defined subset of the nodes of the network. For instance, in designing curative interventions, only some easily accessible proteins are designated as targets for drugs^30^,^31^,^32^. Finally, the need can arise of exerting these control actions without perturbing some nodes that are assigned to particularly important or vital functions. Some of these constraints have been recently considered in ref. 33, where a heuristic strategy is proposed for selecting the driver nodes ensuring controllability of a set of target nodes. However, as stated in ref. 33, a geometrical mapping of this problems is still lacking.

Here, we develop a geometrical framework to comprehensively address the problem of selecting the set of driver nodes that maximizes the diffusion of the control signals through a network in the presence of constraints that inevitably arise in applications. Our method helps the analysis of the readiness of networks to be controllable, allowing us to define the structural permeability of complex networks to control signals, a measure of the extent to which control signals are able to penetrate these networks regardless of the number of driver nodes. Following this approach, we will perform a permeability analysis of both artificially generated and real network topologies. In so doing, we will shed light on certain features that appear to be general, in the sense that they are observed irrespective of the particular network considered.

## Results

### Optimal driver nodes selection

The dynamics of a linear dynamical network formed of *N* nodes is described by


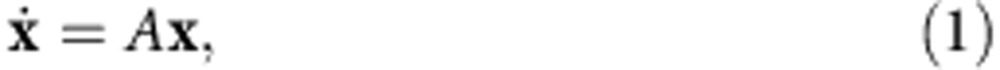


where **x**=[*x*_1_, *x*_2_, ..., *x*_*N*_]^*T*^ is a vector describing the states of the nodes of the network. The matrix *A*={*A*_*ij*_} defines the topology of the network, that is, *A*_*ij*_ measures the coupling from node *j* to node *i*. Roughly speaking, if *A*_*ij*_≠0, then the dynamics 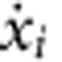 of node *i* depends on that of node *j*. The topology of a network can be represented by means of a graph in which a directed edge connects node *j* to node *i* if the corresponding element *A*_*ij*_ of the matrix *A* is non-zero.

When attempting to control (1), a control signal is injected in a certain number, say *M*, of the nodes. Thus, equation (1) can be rewritten as follows:


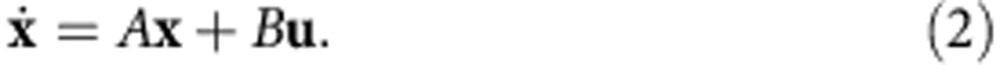


where **u**=[*u*_1_, *u*_2_, ..., *u*_*M*_]^*T*^ is the set of control signals and *B* is an *N* × *M* matrix that reflects the choice of the set of driver nodes Ω_D_. The set of controllable nodes 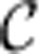, of cardinality 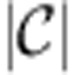, depends on the selection of the set of driver nodes Ω_D_.

According to the structural controllability approach^19^,^34^, the number of controllable nodes of the dynamical network (2) coincides with the generic rank^35^ of its controllability matrix [*B AB A*^2^*B A*^*N*−1^*B*]. In other words, this condition ensures the number of controllable nodes be 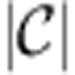 for all values of the non-zero entries of the matrices *A* and *B* except for a set with Lebesgue measure zero. In ref. 34, Hosoe presents a geometrical mapping of the condition on the generic rank of the controllability matrix. Namely, Hosoe states that, given the set of driver nodes Ω_D_, 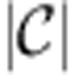 coincides with the dimension of the largest subgraph composed of |Ω_D_| directed paths and disjoint cycles, provided that all nodes of the subgraph must be accessible from the driver nodes (that is, there exists a path from each driver node to each vertex of the subgraph). The dimension of the subgraph is defined as the number of edges it encompasses. A method for the computation of 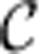, given the set of driver nodes Ω_D_, is given by Poljak^36^. Both Hosoe^34^ and Poljak^36^ aim at evaluating 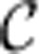, given Ω_D_. In what follows we will show how to optimally choose Ω_D_. In doing so, motivated by the aforementioned economical and physical constraints that in applications limit the ability to freely select the driver nodes, we restrict the selection to a set of admissible nodes, say Ω. Moreover, we assume that controllability is sought of a given set of target nodes, say Φ. We also consider a set of nodes that one does not wish to perturb while exerting the control action, say Ψ, and will refer to these as untouchable nodes. In general, the intersection of the sets Ω and Φ is nonempty, as nodes may act as both targets and admissible drivers. However, the pairs of sets (Φ, Ψ) and (Ω, Ψ) are disjoint.

Our approach allows us to cope with two general problems. In problem 1, we select the set of driver nodes Ω_D_ of fixed cardinality |Ω_D_|=*M* out of the set of the admissible nodes Ω that maximizes 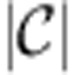, satisfying the constraints that 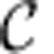 includes the set of target nodes Φ, and that the nodes of the set Ψ are not perturbed. In problem 2, we select the set of driver nodes Ω_D_ of minimum cardinality out of the set of the admissible nodes Ω, satisfying the constraints that 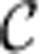 includes the set of target nodes Φ, and that the nodes of the set Ψ are not perturbed.

**Figure 1:**
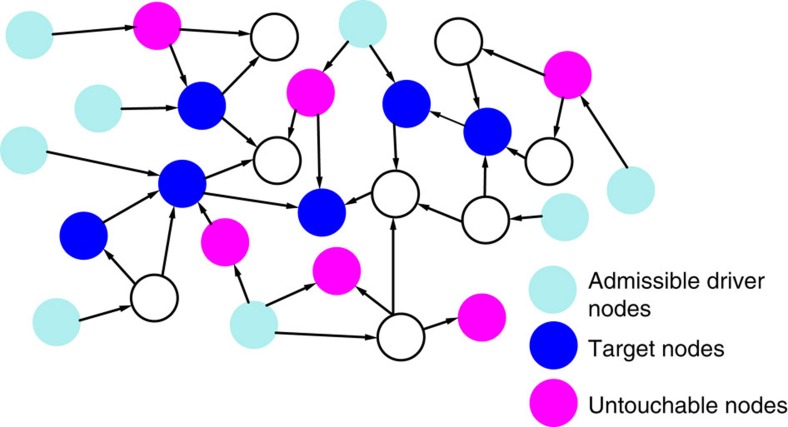
Example of a network to which we will apply our controllability analysis. Turquoise circles represent the nodes of the set Ω, that is, those that can be selected as drivers. Blue circles represent the nodes of the set Φ, that is, those that are targets and must be encompassed in the set 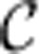. Magenta circles represent the nodes of the set Ψ, that is, those that are untouchable and must not be perturbed by the control action.dfigure.

Figure 1 depicts a typical scenario in which our approach finds application. More information on the formulations of problems 1 and 2 can be found in Supplementary Note 1.

By varying *M* and the composition of the sets Φ, Ψ and Ω, the general problems 1 and 2 can be specified to cope with a broad spectrum of scenarios. Among these, the following are prominent in the literature. If the sets Ω and Φ coincide with the entire set of vertices, Ψ is empty, and there are no constraints on the number of driver nodes, solving problem 2 corresponds to finding the set of driver nodes of minimum cardinality that ensure complete controllability of a network^20^. Moreover, when the set Ω coincides with the entire set of vertices, and the sets Φ and Ψ are empty, solving problem 1 for *M*=1 allows to determine the node with maximum control centrality^37^. We remark that our method yields an improvement with respect to the state of the art, as in ref. 37 the node with maximum centrality is found by an exhaustive application of Hosoe's theorem^34^ to all the nodes of the network. Finally, when Ω coincides with the entire set of vertices, Ψ is empty, and Φ is a well-defined subset of nodes, solving problem 2 allows us to find an optimal solution to the problem for which a heuristic strategy is proposed in ref. 33 (Supplementary Note 1).

Our solution to problems 1 and 2 is based on the following sequence of steps. First, the graph 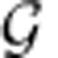 of the network is augmented with additional nodes representing the external control signals to be injected into the network. These are initially connected by means of outgoing and inbounding edges to all nodes of the network. The construction of the augmented graph 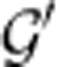 is concluded by adding self-loops to all nodes that do not already have one. The graph 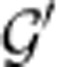 is then partitioned into disjoint cycles. Among all possible cycle partitions of 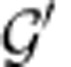, the one is selected that maximizes the number of edges of 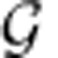 entering nodes accessible from the drivers. This task is performed by solving an integer linear programme (ILP). The set of controllable nodes 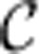 is given by the nodes of the cycle partition of 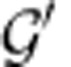 that either have as inbounding edge an edge of 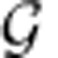, or an edge exiting the nodes that represent the input signals. As the additional edges allow to reduce directed paths to cycles, the optimality of our method is guaranteed by Hosoe's theorem^34^.

**Figure 2:**
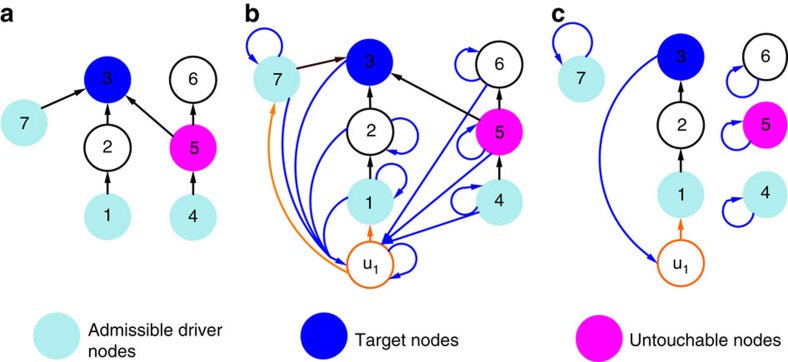
Schematic representation of our method. (**a**) The original graph of the network. (**b**) Construction of the augmented graph. (**c**) Optimal cycle partition returned by the ILP.

An example of application of our method for problem 1 and *M*=1 is shown in Fig. 2; the orange node in Fig. 2b represents the input signal, while the orange and blue edges are those added to enable the formation of a cycle partition. The nodes with inbounding black or orange edges are those encompassed in 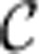. A detailed description of our method for the solution of problems 1 and 2 is given in Supplementary Note 2, along with further details on its optimality.

### Structural permeability of complex networks to control signals

**Figure 3:**
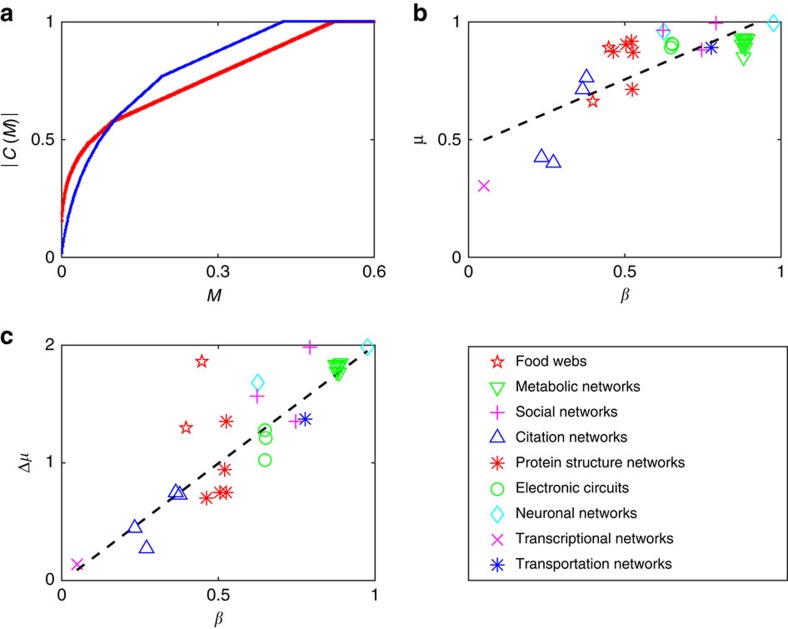
Results of the permeability analysis. (**a**) Plot of 
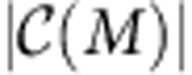
, the number of controllable nodes, over *M*, the fraction of driver nodes deployed, for the Budding Yeast Protein Structure network (red) and the SciNet citation network (blue). (**b**) Plot of the permeability *μ* over *β* for the real networks we have analysed. (**c**) Plot of the loss of permeability caused by the untouchable nodes Δ*μ* over *β* for the real networks we have analysed. In **a** and **b**, the coefficients *β*, *μ* and Δ*μ* are dimensionless.

By solving problem 1 for each value of *M* in the interval of integers [1, *N*] without restrictions on the admissible, target and untouchable nodes, we obtain the sequence of sets of optimal driver node Ω_D_(*M*) and the corresponding 
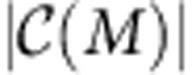
. Figure 3a portrays the sequence 
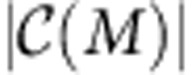
 for the Budding Yeast Protein Structure network (red) and the SciNet citation network (blue), see Supplementary Table 1 for more information on these networks. The question of which one has a greater propensity to be controlled is nontrivial, as, apparently, it varies depending on the value of *M*. Namely, in Fig. 3a, we observe that for small values of *M*, the red curve lies above the blue one, whereas the opposite is observed for *M*>0.1*N*.

To measure the readiness of a network to be controllable, we define the network permeability to control signals *μ*∈[0, 1], which, in the thermodynamic limit, can be computed as


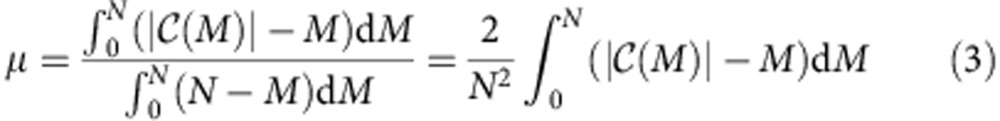


For a given network, *μ* is the difference between the area under the curve 
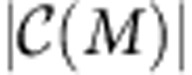
, and the same area relative to an ensemble of *N* disconnected nodes for which 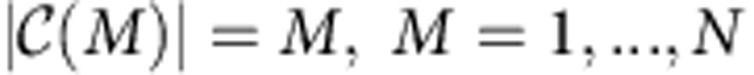. This quantity is then divided by the area under the curve 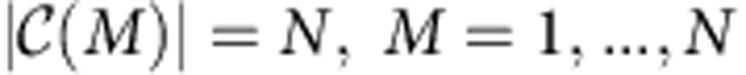 so that *μ* takes the value of 1 for networks that are completely controllable by means of one driver node and the value of 0 for ensembles of disconnected nodes. The integral operator allows *μ* to take into account the dimension of the maximal set of controllable nodes for all values of *M*. We emphasize that while controllability is a property of a network together with the selected driver nodes, *μ* is only related to the network itself. Hence, the permeability allows us to measure, for the first time, the propensity of a network to be controllable, that is, the extent to which the network structure facilitates the diffusion of the control signals. The permeability, as defined above, is a structural network property. To evaluate the extent to which a network can be made controllable in the presence of the sets of admissible, target and untouchable nodes, we define the indexes *μ*(Ω), *μ*(Φ) and *μ*(Ψ) all having values in [−1, 1]. These indexes represent the permeability of a network conditioned to the particular choice of these sets. Further details on *μ*(Ω), *μ*(Φ) and *μ*(Ψ) are given in Supplementary Note 3.

We have analysed a number of real and artificial networks in order to shed light on the relation between their structure and their permeability. As our approach reduces to extracting cycles from a graph, one could expect that networks with high average degree 〈*k*〉 are highly permeable. Surprisingly, we observed that real networks with similar 〈*k*〉 can exhibit large variability in their permeability. Moreover, we find that *μ* is well explained by the parameter


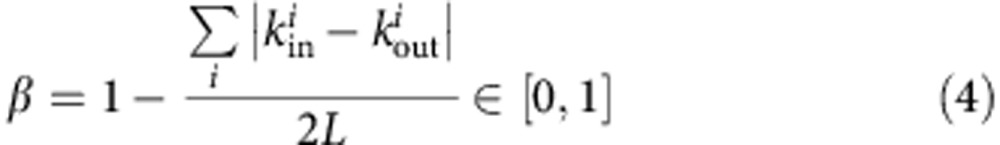


where *L* is the number of edges of the network. The parameter *β* takes the value of 1 for perfectly balanced graphs, that is, graphs for which the indegree *k*_in_ of each node is the same as its outdegree *k*_out_. As shown in Fig. 3b, *β* is correlated (*ρ*=0.79) with *μ*. This is consistent with the theoretical background for our method (provided by Hosoe's theorem^34^) as a cycle partition of a network is a balanced digraph. It is also coherent with the fact that regular networks with 〈*k*〉≥1 and connected undirected networks, both characterized by balanced graphs, can be made controllable by means of only one control signal^20^.

To understand the impact of untouchable nodes, we evaluated the difference between the network permeability *μ* and the conditioned permeability *μ*(Ψ). We found that these impurities tend to jeopardize our ability to control a network, as a small set of untouchable nodes can be responsible for a large loss of permeability. This phenomenon is shown in Fig. 3c, where the correlation (*ρ*=0.81) between *β* and the loss of permeability Δ*μ*=*μ*−*μ*(Ψ) resulting from the addition of untouchable nodes can be appreciated.

**Figure 4:**
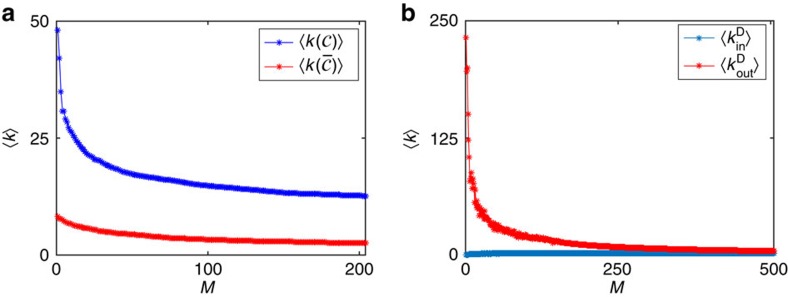
Signature of the controllable and driver nodes. (**a**) Plot of the average degree of the controllable nodes 
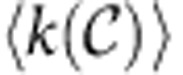
 (blue) and of the non-controllable nodes 
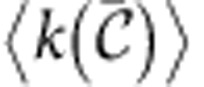
 (red) for the Small World and Griffith citation network. (**b**) Plot of the average indegree 
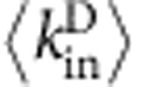
 (blue) and outdegree 
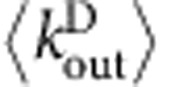
 (red) of the driver nodes versus the number of driver nodes for the Small World and Griffith citation network. In both panels, *M* is the number of driver nodes deployed.

In performing extensive numerical analyses, we kept track of the structural properties of the nodes of the set 
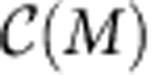
 and of the set of selected driver nodes Ω_D_(*M*) as we varied *M*. We found that both these sets are characterized by signatures. The first observation is that the nodes of 
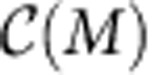
 have higher degree than those of its complement 
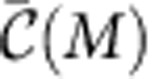
 as shown in Fig. 4a The second observation is that the nodes of Ω_*D*_(*M*) typically exhibit low indegree and high outdegree as shown in Fig. 4b for the Small World and Griffith citation network. We observe that for small values of *M*, nodes with low indegree are selected as drivers. When these are numerous, those with high outdegree are selected first.

These findings have immediate practical relevance. First, the signature of the nodes of 
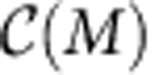
 points out that targeting nodes with low degree requires a large number of drivers, whereas targeting nodes with high degree is feasible with a small set Ω_D_. On the other hand, the signature of the nodes of Ω_D_(*M*) indicates that when lacking the ability to conduct the permeability analysis described above, a good criterion for the selection of the driver nodes is to choose, among the nodes with low indegree, those with high outdegree.

Altogether, our findings provide tools for easily assessing how challenging it is to fulfil given controllability requirements. If a network is characterized by a low value of *β*, the set of admissible nodes does not encompass nodes with low indegree and high outdegree, and the nodes to be targeted have small degree *k*, then even fulfilling the mildest controllability requirements might be a chimera. Conversely, high controllability goals can be achieved when these conditions are not verified.

**Figure 5:**
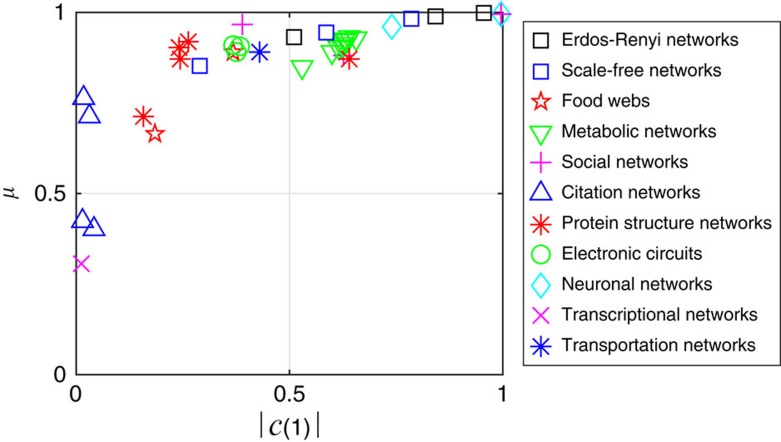
Classification of complex networks. Plot of the permeability *μ* over the maximum centrality 
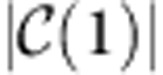
 for model and real networks. The permeability *μ* is dimensionless, while 
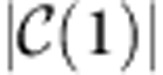
 is the maximum number of nodes controllable with one driver.

Our results show that a large set of controllable nodes can be obtained with a reasonable amount of drivers only when the network structure determines a high permeability. When does this reasonable amount reduce to a handful of nodes, thus knocking down the control costs? We find the answer to this question by classifying networks on the basis of two measures, their permeability *μ* and the maximum centrality 
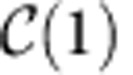
 of their nodes. It follows that networks can be divided into the following three classes (Fig. 5): the first encompassing highly permeable networks (*μ*>0.5) having at least a node with high centrality 
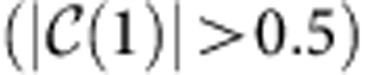
, the second encompassing highly permeable networks that do not have a node with high centrality, and the third class encompassing impermeable networks that do not have a node with high centrality. The networks in the first class are such that a large set of controllable nodes 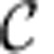 can be obtained inexpensively, that is, by using only a handful of drivers. For networks belonging to the second class, a large 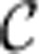 can still be obtained, but at a higher price, as 
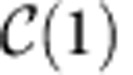
 is small but increases rapidly with the number of drivers. Finally, networks in the third class tend to be impermeable to control signals regardless of *M*. For these networks, only mild contollability requirements can be fulfilled and at a high price, and the role played by the sets of target, admissible and untouchable nodes is critical. Figure 5 also shows that the topology of networks that share the same functions (in particular, protein networks, metabolic networks and electric circuits) tends to fall in the same class.

## Discussion

In general, the control of a complex dynamical network can be performed in several ways by selecting, among its nodes, the ones in which the control signals are injected. When the number of driver nodes is limited by practical constraints, the problem arises of how to select them in order to allow the control signals to permeate through the network as deeply as possible. By taking this new perspective, we develop a geometrical mapping of this problem for large linear dynamical networks with arbitrary connectivity. This mapping allows us to find the optimal selection of the driver nodes by solving an ILP in which constraints on driver, target and untouchable nodes can also be considered. Leveraging the possibility of solving such a problem for increasing numbers of drivers, we gain insight in how the topological structure of a network can facilitate or prevent the diffusion of the control signals. To measure the structural propensity of a network to be controlled, in part or in toto, we introduce the structural permeability of complex networks to control signals.

Overcoming the framework of complete controllability in favour of limited and well-defined controllability goals, enables us to numerically investigate the permeability of a number of both real and synthetic networks, by also taking into account the role of driver, target and untouchable nodes. We observe that the network permeability well correlates with an easily computable network topological parameter and we are able to extract signatures that characterize both the selected driver nodes and those in the controllable set. Finally, on the basis of the permeability index and the control centrality, we propose a taxonomy of all the networks considered in the paper.

## Methods

### Analysis of artificial topologies

Scale-free topologies were generated by means of the directed version of the so-called static model^38^. All numerical results on artificial networks are averaged over 100 topologies of 1,000 nodes each.

### Analysis of real networks

A complete list of the topologies of real networks analysed is available in Supplementary Note 4. The results of the evaluation of the loss of permeability in the presence of untouchable nodes are relative to a random selection, for each real network analysed, of 5% of its nodes to be untouchable. Results are averaged over 50 different random selections of untouchable nodes per network. We also used slightly different selection criteria, varying the size of the set Ψ and the average degree of its nodes, and recorded qualitatively similar results as detailed in Supplementary Note 5. For networks of finite dimensions, *μ* has been computed approximating the integral (3) by means of the trapezoidal method.

### ILP solver

The numerical analysis was conducted on the Matlab platform by using the ILP solver intlinprog. We emphasize that the method we propose can be implemented also on other available commercial software.

## 

## Additional information

**How to cite this article:** Lo Iudice, F. *et al*. Structural permeability of complex networks to control signals. *Nat. Commun.* 6:8349 doi: 10.1038/ncomms9349 (2015).

## Supplementary Material

Supplementary Information Supplementary Figures 1-3, Supplementary Table 1, Supplementary Notes 1-5 and Supplementary References.
