# Supplementary material for: Structural permeability of complex networks to control signals
Source: Nat Commun. 2015 Sep 22;6:8349. doi: 10.1038/ncomms9349 (PMC4595749; doi:10.1038/ncomms9349)
Supplement: Supplementary Information — Supplementary Figures 1-3, Supplementary Table 1, Supplementary Notes 1-5 and Supplementary References. [file ncomms9349-s1.pdf]

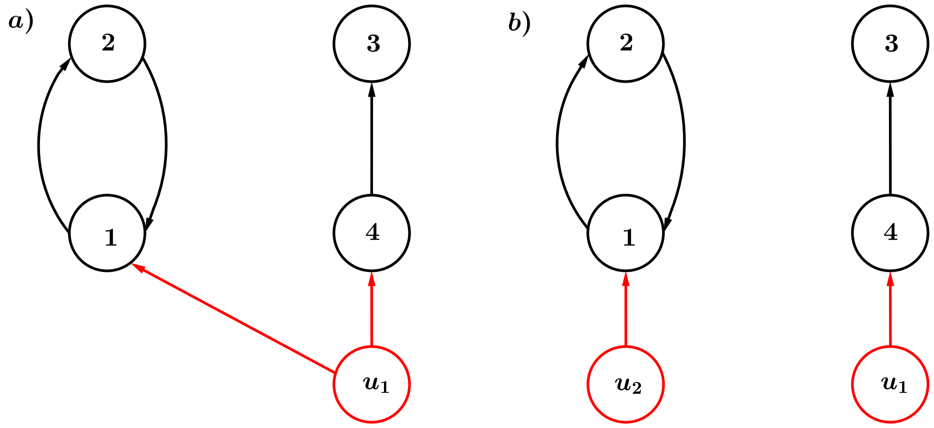

Supplementary Figure 1: Minimal set of driver nodes according to the definition in [2] (left), and according to the definition considered in this paper(right).

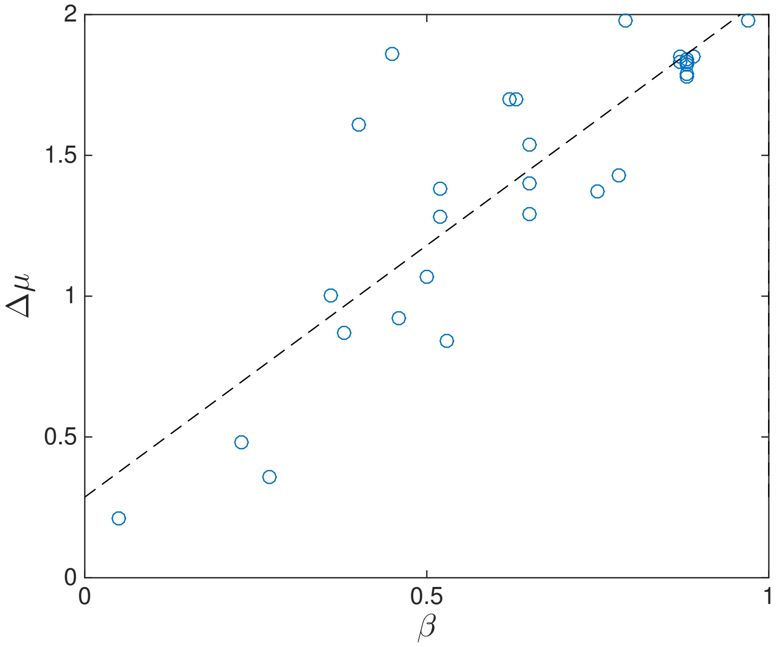

Supplementary Figure 2: Plot of the loss of permeability $\Delta\mu$ versus β in the presence of a randomly chosen set of untouchable nodes of cardinality equal to $0.1N$ of the nodes of the network.

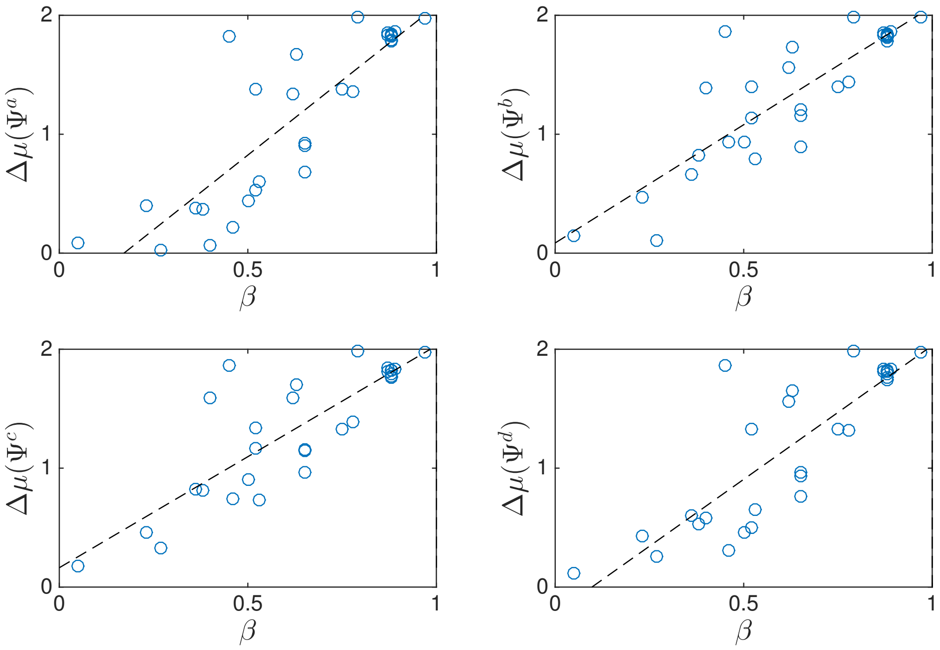

Supplementary Figure 3: Plot of the loss of permeability $\Delta\mu(\Psi^i)$ versus β in the presence of untouchable nodes chosen according to criteria a) through d).

Network	$\lvert\mathcal{C}(1)\rvert$	μ	N	β
Aeropyrum pernix Metabolic[7]	0.63	0.91	1057	0.88
Air Traffic Control [8]	0.43	0.89	1226	0.78
B cell Interactome [9]	0.64	0.87	5737	0.52
Budding Yeast [10]	0.16	0.71	2361	0.53
C. Elegans Metabolic [7]	0.62	0.91	1173	0.88
C. Elegans Neuronal I[11]	0.74	0.96	306	0.63
C. Elegans neurnoal II[12]	0.99	0.99	281	0.97
DBLP Citation [13]	0.01	0.42	12591	0.23
E. Coli Metabolic [7]	0.53	0.85	2275	0.88
Emericella Nidulans Metabolic [7]	0.63	0.92	916	0.88
Florida food web[10]	0.37	0.89	128	0.45
Little Rock Lake food web[13]	0.19	0.66	183	0.40
Mycobacterium Tubercolosis [7]	0.62	0.90	1520	0.87
Mycoplasma Pneumoniae Metabolic [7]	0.63	0.93	411	0.87
OC link social [11]	0.63	0.88	1899	0.75
Physicians Trust [14]	0.39	0.97	246	0.62
Protein Structure I[15]	0.26	0.92	95	0.52
Protein Structure II[15]	0.25	0.87	53	0.46
Protein Structure III[15]	0.24	0.90	99	0.50
Residence Hall Social [13]	0.99	0.99	217	0.79
s208 Electronic Circuit [15]	0.38	0.89	122	0.65
s420 Electronic Circuit [15]	0.39	0.91	252	0.65
s838 Electronic Circuit [15]	0.37	0.91	512	0.65
S. Cerevisiae Metabolic [7]	0.60	0.89	1511	0.88
Scientometrics Citation [10]	0.02	0.76	2729	0.38
Small World Citation [10]	0.04	0.40	395	0.27
Small World & Griffin citation [10]	0.03	0.71	1024	0.36
Thermotoga Maritima Metabolic [7]	0.66	0.93	830	0.89
Treponema Pallidum Metabolic [7]	0.64	0.93	485	0.88
TRN-yeast[15]	0.01	0.30	688	0.05

Supplementary Table 1: List of real network topologies analysed.

Preliminaries

In what follows, we make use of the following definitions from [1]:

Definition 1 *A stem is an elementary path i.e., a sequence of oriented edges* $\{(p_{ij}), (p_{jk}), ..., (p_{l,m})\}$ *such that* $i \neq m$. *We will name the start node* i *a source, and the end node* m *a sink.*

Definition 2 *A cycle is an an elementary path that starts and ends in the same node.*

Moreover, we denote by $G(\mathcal{V}, \mathcal{P})$ the graph of a dynamical network, with $\mathcal{V}$ being the set of vertices of G and $\mathcal{P}$ the set of edges. If $p_{ij} \in \mathcal{P}$ then an edge connects node j to node i and the corresponding element A_{ij} of the matrix A is nonzero.

Definition of a driver node: differences with the existing literature Most of the recent literature on structural controllability of complex networks implicitly or explicitly relies on the definition of *driver nodes* given in [2]. In [2] the number of driver nodes coincides with the number of different input signals injected in a network. Following this definition the number of nodes in which a control signal can be injected is either larger than or equal to the number of control signals, as the same signal can be injected in different nodes. As an example, take the simple network represented in black in Supplementary Figure 1a). This network is controllable by means of only one input signal (the red node u_1 in Supplementary Figure 1a)), but requires injecting the input signal into two different nodes, as the cycle is disjoint from the stem.

Here, we take a different perspective: a *driver node* is any node of the network which has been selected as an access point (a gate) and from which a control signal can permeate through the network (nodes 1 and 4 in Supplementary Figure 1a)). The reason why we we use this different definition is that usually, in applications, limitations are imposed on the number of nodes in which a signal is injected, rather than on the number of signals. Moreover, from an energetic standpoint, injecting the same signal into two different nodes may be inefficient as the same control action is used to achieve a larger number of objectives. To better understand this point consider the problem of steering the linear dynamical system

$$\dot{\mathbf{x}} = A\mathbf{x} + B\mathbf{u} \tag{1}$$

where $\mathbf{u}(t) = [u_1(t)\ u_2(t)]^T$, from an initial state $\mathbf{x}(0)$ to a desired final state $\mathbf{x}(t_1)$. In order to minimize the energy required to achieve the control goal,

$\mathbf{u}(\mathbf{t})$ is chosen as the solution of the following optimization problem:

$$\min_{\mathbf{u}(\mathbf{t})} J := \int_0^{t_1} \mathbf{u}(\mathbf{t})^T \mathbf{u}(\mathbf{t}) dt, \tag{2}$$

As imposing $u_1(t) = u_2(t)$ represents a constraint for the optimization problem in (2), bounding two signals to be identical can only yield an increase in the optimal J.

An important effect of our definition of a driver node is that we are able to completely characterize the *structure* of the matrix B, that is the position of its nonzero entries. In our framework, each column of B is a versor, *i.e.*, each of its columns contains a single non-zero entry in correspondence of the associated driver node. This information is not provided by the methods that use the definition of driver nodes given in [2] for which, fixing the number of control signals only fixes the number of columns of B, not the number of its nonzero entries. As an example, consider again the network in Supplementary Figure 1*a*). According to the maximal matching algorithm, only one driver node (intended as an input signal as in [2]) is required to gain full control of the network as the only unmatched node is 4. Nevertheless the control signal u_1 needs to be injected also in either node 1 or 2, an information that the maximal matching algorithm does not provide.

While our method for the selection of the driver nodes detailed in Supplementary Note 2 has been developed based on our definition of a driver node, we will show that it can also be used when the definition of a driver node given in [2] is used, thus preserving the generality of our approach.

Supplementary Note 1: Problem Formulation

Here we give a formal statement of the two general problems introduced in the main text and we show how these can be specified to cope with a number of relevant scenarios. We start by considering Problem 1, which can be formally stated as follows:
Problem 1:

$$\max_{\Omega_D \subset \Omega} |\mathcal{C}| \tag{3}$$

subject to

$$|\Omega_D| = \mathrm{M} \tag{4}$$

$$\Phi \subset \mathcal{C} \tag{5}$$

$$\cup_{i \in \Psi} (\Xi_i) \cap \Omega_D = \emptyset, \tag{6}$$

where Ω is the set of admissible nodes, Φ is the set of target nodes, $\mathcal{C}$ is the set of controllable nodes, Ξ_i is the set of nodes from which each untouchable node i in Ψ is accessible, and Ω_D is the set of driver nodes of cardinality M to be selected. This general formulation allows us to deal, as particular cases, with the following two relevant problems.
a. Finding the node with maximum centrality When (i) the set Ω coincides with the entire set of vertices of $G(\mathcal{V}, \mathcal{P})$ and (ii) the sets Φ and Ψ are empty, solving Problem 1 for $M = 1$ allows us to determine the node with maximum control centrality [3].
b. Finding the largest set of controllable nodes given the set of driver nodes (Hosoe's theorem) When (i) the set Ω_D is given and (ii) the sets Φ and Ψ are empty, solving Problem 1 allows us to determine the set of controllable nodes, given the set of driver nodes. Note that in this scenario, our strategy reduces to Poljak's [4] method as the set Ω_D is given and no driver node has to be selected.
We now give a formal statement of Problem 2,
Problem 2:

$$\min_{\Omega_\mathrm{D} \subset \Omega} |\Omega_\mathrm{D}| \tag{7}$$

subject to

$$\Phi \subset \mathcal{C} \tag{8}$$

$$\cup_{i \in \Psi} (\Xi_i) \cap \Omega_\mathrm{D} = \emptyset. \tag{9}$$

Again this general formulation allows us to cope, as particular cases, with two prominent problems.
a. Finding the MDS of a network If (i) the sets Ω and Φ coincide with the entire set of vertices and (ii) Ψ is empty, solving Problem 2 corresponds to finding the set of driver nodes of minimum cardinality that ensure complete controllability of a network [2].
b. Target Controllability When (i) Ω coincides with the entire set of vertices, (ii) Ψ is empty, and (iii) Φ is a well defined subset of nodes, solving Problem 2 allows to find an optimal solution to the problem for which a heuristic strategy is proposed in [6].

Supplementary Note 2: Problem solution

The solution to the optimization problems stated in the previous Section is obtained by transforming them in corresponding problems on a graph.

To illustrate this, we start by considering Problem 1 whose solution requires performing two preliminary steps. First, to fulfill the requirement on

the untouchable nodes, we restrict the selection of the driver nodes to the set Ω' of nodes that do not have a vertex of Ψ in their downstream. Namely, we have that $\Omega' = \{\Omega - \cup_{i|v_i \in \Psi} \Xi_i\}$ where each set Ξ_i encompasses all the vertices from which node v_i is accessible. Then, we build a new *weighted augmented* graph $\mathcal{G}'$ in which the M input signals to be injected in the network are represented as additional nodes belonging to a set Θ. These are initially connected to each node of Ω' with edges of unit weight, and have zero weight edges coming from each node of G. The edges exiting the nodes of Θ allow the inputs to be injected in any of the nodes of Ω', while the inbounding edges allow cycles to be closed in the nodes of Θ. Finally, self loops of zero weight are added to each node of G' except for the nodes of Θ and for the target nodes. The self loops allow each non-driver and non-target node to form an independent cycle. All the edges already existing in the graph G are taken with unit weight. This new augmented weighted graph will be indicated by $G'(\mathcal{V}', \mathcal{P}', \mathcal{W}')$.

Problem 1 can now be translated into a problem on the graph G'. We must *select, among all the partitions of G' in disjoint cycles, the one that encompasses the maximum number of edges with unit weight and satisfies the following constraint: the nodes in cycles encompassing edges with unit weight must be accessible (in $\mathcal{G}(\mathcal{V}, \mathcal{P})$) from the driver nodes.*

This problem translates into the following Integer Linear Program (ILP)

$$\max_{y} \quad \sum_i \sum_j w'_{ij} y_{ij} \tag{10}$$

subject to

$$y_{ij} \in \{0, 1\} \quad \forall i, j | p'_{ij} \in \mathcal{P}' \tag{11}$$

$$\sum_j y_{ij} = 1 \quad \forall i = 1, ..., N + M \tag{12}$$

$$\sum_i y_{ij} = 1 \quad \forall j = 1, ..., N + M \tag{13}$$

$$\sum_{l \in \Xi_i} \sum_{j \in \Theta} y_{lj} \geq \sum_j w'_{ij} y_{ij} \quad \forall i = 1, ..., N + M \tag{14}$$

Here, a binary decision variable y_{ij} is associated with each edge p'_{ij} of the graph G'; if $y_{ij} = 1$, then the corresponding edge p'_{ij} will be part of the cycle partition. The product $w'_{ij} y_{ij}$ will return a unit cost only either when the selected edge of G' is also an edge of G or if the edge exits a node of Θ. Every other selected edge (needed to form a cycle) will not contribute to the objective function. Hence, (10) represents the maximal achievable cardinality of the set of controllable nodes $|\mathcal{C}|$. The nodes of the cycle partition with an

inbounding edge of unit weight compose the set $\mathcal{C}$. The role of the constraints of the ILP is that of ensuring the solution fulfill the requirements of Problem 1. Eqs. (12) and (13) guarantee that the optimal solution be a cycle partition of G' by forcing each one of its vertices to have exactly one inbounding and one outgoing edge, while Eq. (14) forces all the nodes of $\mathcal{C}$ to be accessible from at least one of the driver nodes.

Proceeding this way, a node is part of $\mathcal{C}$ if it is either (i) a driver node, or (ii) part of a cycle originating from a driver node, or (iii) part of a cycle with edges of unit weight accessible from a driver node. Note that the constraint on the target nodes is automatically fulfilled as these do not have self loops and hence must have an inbounding edge of unit weight. By removing the edges with zero weight from the optimal cycle partition along with the resulting isolated nodes, we obtain the largest stem-cycle disjoint subgraph of $\mathcal{G}$ originating from M driver nodes. Hence, the optimality of our method is a direct consequence of Hosoe's theorem [5].

Coping with Problem 2 requires only minor tweaks to the method described above. As the number of driver nodes is now to be determined and is bounded by the cardinality of Ω', $|\Omega'|$ new nodes must be added to the graph $\mathcal{G}$ in the construction of the graph $\mathcal{G}'$ and a self loop must be added to each of these nodes. Furthermore, the objective function of the ILP (Eq. (10)) must now read

$$\min_{y_{ij}|v_j\in\Theta} \sum_{j|v_j\in\Theta} \sum_{i} w'_{ij} y_{ij}. \tag{15}$$

Thus, in this case, a cycle partition is sought that minimizes the number of driver nodes necessary to fulfill the requirement on the target and untouchable nodes. The vertices representing input signals that are not injected in the network form cycles of their own thanks to the addition of the self loops.

We conclude the discussion of our method by remarking that in order to adapt our method to the definition of a driver node given in [2], the constraint in Eq. (14) must be removed from the ILP.

Optimality of our method As anticipated, our method for the solution of Problem 1, seeks to find the largest stem-cycle disjoint subgraph originating from M driver nodes. Hence, its optimality is a direct consequence of Hosoe's theorem. This is evident if we reformulate Problem 1 as a set of two nested optimization problems

$$\max_{\Omega_\mathrm{D}\subset\mathcal{V}} \left[\max\ |\mathcal{C}| \mid \Omega_\mathrm{D}\right] \tag{16}$$

From a conceptual standpoint the internal maximization problem in Eq. (16) corresponds to finding, for all possible sets of M driver nodes, the largest

stem cycle disjoint subgraph, while the external maximization problem in Eq. (16) corresponds to selecting the set of driver nodes that generates the largest subgraph among those resulting from the internal problem. This combinatorial problem is mapped into an ILP by means of the steps described in section Supplementary Note 2.

The optimality of Problem 2 follows from an analogous argument. Namely, for all possible $\Omega_{\mathrm{D}} \subset \Omega'$ of arbitrary cardinality the stem-cycle disjoint subgraph is sought that encompasses all of the target nodes. Then amongst all the sets of driver nodes that allow the formation of a stem-cycle disjoint subgraph, we select the one of minimum cardinality.

Feasibility The paper relies on the implicit assumption that our method is applied to a well formulated problem, *i.e.,* a problem that admits a solution. However, this would not be the case, for instance, if an untouchable node were in the downstream of a target node, as for the latter to be controllable, the former must be influenced. We remark that the feasibility issues are not related to our method but to the problem itself. Hence, if our method does not output a solution, then a solution to the problem does not exist in the structural controllability framework.

Computational Considerations We start the discussion of the computational aspects of our problem by providing a stronger formulation of the ILP in (10)-(14). To obtain the strengthened formulation, we build $\mathcal{G}'$ by adding only one node representing all the input signals. This node, denoted as $N+1$, now plays the role of all the nodes of Θ and is connected to all of the other N nodes of $\mathcal{G}'$ with both inbounding and outgoing edges. Node $N+1$ has the ability to *close* all of the M cycles that include a driver node. Coherently, the equality constraints in Eqs. (12) and (13) are changed to

$$\sum_j y_{ij} = 1 \quad \forall i = 1, ..., N \tag{17}$$

$$\sum_i y_{ij} = 1 \quad \forall j = 1, ..., N \tag{18}$$

$$\sum_j y_{N+1,j} = M \tag{19}$$

$$\sum_j y_{j,N+1} = M. \tag{20}$$

The advantage of this new formulation is that it considerably reduces both the number of variables and the number of constraints. It is worth noting that in both formulations the equality constraint matrix is totally unimodular. As we will show in what follows, this structural property can be exploited to strongly reduce the computational complexity of our ILP.

The general strategy for effectively solving the problem (10) - (14) is that of complementing the general *Branch and Cut* techniques used to solve ILPs with ad hoc *bounding* and *cutting* procedures able to exploit the special structure of our problem.
Bounding: estimation of an upper bound for $|\mathcal{C}|$. An approximation of $|\mathcal{C}|$ from above can be obtained from a relaxed version of our problem.
<u>Relaxation A:</u> a first relaxation can be obtained, as usual, by removing the integrity constraints on the decision variables y_{ij} thus transforming the ILP into an LP. Note that as the equality constraint matrix is totally unimodular often this relaxation will yield an integer solution allowing the ILP to stop at the root LP solution.
<u>Relaxation B:</u> a second strategy is that of relaxing constraint (14). In this case the problem becomes that of finding the cycle partition of $\mathcal{G}'$ that maximizes our objective function. As the equality constraint matrix is totally unimodular, also this problem reduces to a LP. Obviously, in bounding from above the objective function of problem (10) - (14) the minimum between the two estimates can be selected.
Bounding: estimation of a lower bound for $|\mathcal{C}|$. An approximation from below of the number of controllable nodes can be obtained from Relaxation B. Indeed, the solution of the relaxed problem offers a cycle covering of the graph G' for which not necessarily each cycle is accessible from the drivers. By subtracting the number of nodes located in inaccessible cycles to the objective function we get the approximation required.

Ad hoc Cutting procedures The branching phase in ILP solution methods is always complemented with a *cutting* procedure. Cuts are additional constraints added to the ILP that allow strengthening the formulation without eliminating feasible solutions. In our case, if the upper bound resulting from Relaxation A is tighter, then standard cuts (Gomory, Strong, etc.) and binary branching strategies are to be applied (although always taking into account the availability of a lower bound). On the other hand, if the upper bound obtained by relaxing the constraints in (14) is tighter, then two *ad hoc* cutting strategies can be deployed *which exploit* the structure of our problem. Both consist in applying recursively some constraints.
Strategy 1: The first ad hoc cutting strategy consists of the following steps:

1. Relax the constraints in Eq. (14) and solve the LP relaxation of the resulting ILP. The solution will be integer as the equality constraint matrix is totally unimodular. This yields a set of driver nodes Ω_{D}^1 and a set of controllable nodes $\mathcal{C}^1$;

2. If each node of $\mathcal{C}^1$ is accessible from at least one node of Ω_{D}^1 then the optimal solution is found;

3. Otherwise, $|\mathcal{C}^1|$ is an upper bound for the optimal value $|\mathcal{C}^*|$;

4. Remove the inaccessible nodes from $\mathcal{C}^1$ to obtain a lower bound for $|\mathcal{C}^*|$ along with the largest set of controllable nodes from the set of drivers Ω_{D}^1;

5. Apply the inequality constraint

$$\sum_{l \in \Omega_{\mathrm{D}}^1} \sum_{j \in \Theta} y_{lj} \leq M - 1 \tag{21}$$

to exclude the set Ω_{D}^1 from the possible sets of drivers and solve the LP relaxation of the new ILP.;

6. If the solution is integer but unfeasible as some cycles are inaccessible, two new bounds can be computed as in steps 3 and 4. Take the tightest among the available bounds and repeat steps 5 and 6 until either an integer feasible solution or a non-integer feasible solution is found;

7. If an integer feasible solution is found, then the optimal solution is the best one between such solution and the current lower bound;

8. If on the other hand a non-integer solution is found, then this is the tightest possible upper bound. Now, the standard cuts and branching strategies must be applied (taking into account the availability of upper and lower bounds to enhance the pruning procedure).

Strategy 2: The first four steps of the second *ad hoc* cutting strategy coincide with the first four steps of *Strategy 1.* These must be complemented by the following steps;

5. Amongst the inequality constraints in (14) apply only those violated by the solution obtained at step 1;

6. If the solution is integer but unfeasible, as some cycles are inaccessible, two new bounds can be computed as in steps 3 and 4. Take the tightest among the available bounds and repeat steps 5 and 6 until either an integer feasible solution or a non-integer feasible solution is found;

7. If the solution is integer and feasible, then it is optimal;

8. If the solution is non-integer and feasible, then this is the tightest possible upper bound. Now, the standard cuts and branching strategies must be applied (taking into account the availability of upper and lower bounds to enhance the pruning procedure).

The idea behind both cutting strategies is that the sequence of upper and lower bounds obtained by exploiting the total unimodularity of the equality constraint matrix can drive the search toward the true solution. If the search is stopped before the solution is found (because it exceeds the maximum time, for instance) an interval of uncertainty on the number of controllable nodes is obtained. This provides an approximation of the optimal solution and, if necessary, allows to obtain a tight estimate of the permeability of a network.

Supplementary Note 3: Conditioned Permeability

The network permeability μ, defined in Eq. (3) of the main text, is a property of the network structure. Coherently, μ is computed without taking into account the role played by the sets Ω, Φ, and Ψ, and takes the limit value of 0 for an ensemble of disconnected nodes, as the diffusion of signals is prevented by the absence of connectivity. This corresponds to the worst case scenario in terms of permeability. Nevertheless, when the sets Ω and Ψ are considered, some nodes of the network become inaccessible directly and indirectly. In such case the (approximate) computation of the integral in Eq. (3) of the main text can yield negative values. While this may seem incoherent, it well captures the fact that the constraints on the admissible and untouchable nodes can lead to a network being less propense to being controllable than an ensemble of disconnected nodes, as some parts of the network become inaccessible by the control signals. To capture the impact of the sets Ω, Φ, and Ψ on the network permeability μ, we introduce the conditioned permeabilities $\mu(\Omega)$, $\mu(\Phi)$, and $\mu(\Psi)$ which are defined in the interval $[-1, 1]$. We would like to stress that these indexes are properties of both the network and of the specific control problem that is addressed.

The conditioned permeability is computed by means of the same formula given in equation (3) of the main text where N is still the number of nodes of the network and M is the number of driver nodes deployed. The only difference is that $\mathcal{C}(M)$ is the result of an optimization problem where either the constraint on the untouchable, target, or admissible nodes is considered depending on whether we are computing $\mu(\Psi)$, $\mu(\Phi)$, or $\mu(\Omega)$, respectively. To compute the integral in (3) when the set Ω' does not coincide with the entire set of nodes of a network (which is the case when in the presence of admissible and untouchable nodes), one must set $|\mathcal{C}(M)| = |\mathcal{C}(|\Omega'|)|$ for all values of M greater than $|\Omega'|$.

Supplementary Note 4: List of real network topologies analyzed

In Supplementary Table 1 we give the complete list of real network topologies analyzed, along with their maximal centrality $|\mathcal{C}(1)|$, structural permeability to control signals μ, and loss of permeability resulting from a random choice of $0.05N$ untouchable nodes. The results relative to the loss of permeability in the presence of untouchable nodes are averaged over 50 different random choices of the latters for each network.

Supplementary Note 5: Permeability Conditioned to the presence of untouchable nodes

The results shown in Figure 3c of the main text on the effect of the untouchable nodes are for a random extraction of $0.05N$ nodes to be included in the set Ψ. We have conducted the same analysis also under different selection criteria obtaining similar results, which provide further support to our claims. For example, if the cardinality of the set Ψ is increased to $0.1N$ of the network nodes, the loss of permeability $\Delta\mu$ correlates even better with β ($\rho = 0.86$) as can be noticed in Supplementary Figure 2.
We also performed an additional set of analyses in which we reverted to a cardinality of $0.05N$ untouchable nodes but considered four different selection criteria for the set Ψ. We randomly chose the untouchable nodes from

a) the nodes with low indegree (nodes with degree lower than the 33-rd percentile of the indegree distribution);

b) the nodes with low outdegree (nodes with degree lower than the 33-rd percentile of the outdegree distribution);

c) the nodes with high indegree (nodes with degree higher than the 67-th percentile of the indegree distribution);

d) the nodes with high outdegree (nodes with degree higher than the 67-th percentile of the outdegree distribution);

For each criterion and for each topology of a real network in our database, we performed 50 selections of the set Ψ and averaged the resulting conditioned permeabilities. We found that these differences are substantially smaller than the loss of permeability in the presence of a completely random extraction

of the set Ψ. Specifically, we found that

$$\left\langle \frac{(\Delta\mu(\Psi^a) - \Delta\mu(\Psi^r))^2}{(\Delta\mu(\Psi^r))^2} \right\rangle = 0.12,$$
$$\left\langle \frac{(\Delta\mu(\Psi^b) - \Delta\mu(\Psi^r))^2}{(\Delta\mu(\Psi^r))^2} \right\rangle = 0.02,$$
$$\left\langle \frac{(\Delta\mu(\Psi^c) - \Delta\mu(\Psi^r))^2}{(\Delta\mu(\Psi^r))^2} \right\rangle = 0.01,$$
$$\left\langle \frac{(\Delta\mu(\Psi^d) - \Delta\mu(\Psi^r))^2}{(\Delta\mu(\Psi^r))^2} \right\rangle = 0.04,$$

where:

- μ is the permeability of the network;
- $\Delta\mu(\Psi^i) = \mu - \mu(\Psi^i)$ is the permeability conditioned to the presence of untouchable nodes selected according to the i-th criterion;
- $\Delta\mu(\Psi^r) = \mu - \mu(\Psi^r)$ is the permeability conditioned to the presence of completely randomly selected untouchable nodes (the criterion used in the main text of the paper);
- $\langle \cdot \rangle$ indicates that the quantities are averaged over the networks in our databases and over 50 different extractions of the sets Ψ^i.

Moreover, Supplementary Figure 3 shows that the correlation between the loss of permeability $\Delta\mu(\Psi^i)$ and β, when applied to all of the networks in our dataset, is confirmed, irrespective of the specific selection criterion considered. In fact, $\Delta\mu(\Psi^a)$, $\Delta\mu(\Psi^b)$, $\Delta\mu(\Psi^c)$, and $\Delta\mu(\Psi^d)$ have a correlation with β of 0.86, 0.86, 0.84, and 0.85, respectively.

Supplementary References

[1] Lin, C.-T. Structural controllability. *Automatic Control, IEEE Transactions on* **19,** 201-208 (1974).

[2] Liu, Y.-Y. Slotine, J.-J. & Barabasi, A.-L. Controllability of complex networks. *Nature* **473,** 167-173 (2011).

[3] Liu,Y.-Y. Slotine, J.-J. & Barabasi, A.-L. Control centrality and hierarchical structure in complex networks. *PLoS ONE* **7,** e44459 (2012).

[4] Poljak, S. On the generic dimension of controllable subspaces. *Automatic Control, IEEE Transactions on* **35,** 367-369 (1990).

[5] Hosoe, S. Determination of generic dimensions of controllable subspaces and its application. *Automatic Control, IEEE Transactions on* **25,** 1192-1196 (1980).

[6] Gao, J. Liu, Y.-Y. D'Souza, R. M. & Barabási, A.-L. Target control of complex networks, *Nature communications* **5,** 5415 (2014).

[7] Barabasilab dataset of metabolic networks. Available at: *http://www3.nd.edu/ networks/resources/metabolic/index.html*

[8] Ma'ayan Laboratory Network Dataset. Available at: *http://research.mssm.edu/maayan/datasets*

[9] Califano lab interactome datasets, Available at: *http://wiki.c2b2.columbia.edu/califanolab*

[10] Pajek's dataset Available at: *http://vlado.fmf.uni-lj.si/pub/networks/data*

[11] Tore Opsahl dataset. Available at: *http://toreopsahl.com/datasets/*

[12] Wormatlas dataset. Available at: *http://www.wormatlas.org*

[13] Konect Dataset: Available at: *http://konect.uni-koblenz.de/networks*

[14] Freeman's dataset. Available at: *http://moreno.ss.uci.edu/data.html#ckm*

[15] Uri Alon lab dataset. Available at: *http://www.weizmann.ac.il*
